# Clinical criteria for Mucosal Leishmaniasis diagnosis in rural South America: A systematic literature review

**DOI:** 10.1371/journal.pntd.0010621

**Published:** 2022-08-10

**Authors:** Jacob M. Bezemer, Kevin Meesters, Cristhian L. Naveda, Paulo R. L. Machado, Manuel Calvopiña, Mariska M. G. Leeflang, Henk D. F. H. Schallig, Henry J. C. de Vries

**Affiliations:** 1 Fundación Misión Cristiana de Salud, Shell, Pastaza, Ecuador; 2 Department of Medical Microbiology and Infection Prevention, Laboratory for Experimental Parasitology, Amsterdam UMC location University of Amsterdam, Amsterdam, the Netherlands; 3 Amsterdam Institute for infection and Immunity (AII), Infectious Diseases Program, Amsterdam, the Netherlands; 4 Department of Pediatrics, Division of Infectious Diseases, University of British Columbia, Vancouver, Canada; 5 Serviço de Imunologia, Hospital Universitário Prof. Edgard Santos, Universidade Federal da Bahia, Salvador, Bahia, Brazil; 6 OneHealth Research Group, Carrera de Medicina, Facultad de Ciencias de la Salud, Universidad de las Americas, Quito, Ecuador; 7 Department of Epidemiology and Data Science, Amsterdam UMC location University of Amsterdam, Amsterdam, the Netherlands; 8 Department of Dermatology, Amsterdam UMC location University of Amsterdam, Amsterdam, the Netherlands; 9 Center for Sexual Health, Department of Infectious Diseases, Public Health Service, Amsterdam, the Netherlands; Federal University of Ceará, Fortaleza, Brazil, BRAZIL

## Abstract

**Background:**

Mucosal Leishmaniasis (ML), a neglected tropical disease caused by *Leishmania* parasites, impairs the quality of life of under-resourced populations in South America. If not treated promptly, this disease progresses to facial deformities and death. The low sensitivity of microscopy results and the unavailability of other accurate tests hamper the diagnosis. As clinical criteria are readily available in any setting, these may be combined in a syndromic algorithm, which in turn can be used as a diagnostic tool. We explore potential clinical criteria for a syndromic diagnostic algorithm for ML in rural healthcare settings in South America.

**Methodology/Principal findings:**

The protocol for this systematic review was pre-registered in PROSPERO with the number: CRD42017074148. In patients with ML, described in case series identified through a systematic retrieval process, we explored the cumulative ML detection rates of clinical criteria. Participants: all patients with active mucosal disease from an endemic area in South America. Any original, non-treatment study was eligible, and case reports were excluded. PUBMED, EMBASE, Web of Science, SCIELO, and LILACS databases were searched without restrictions. The risk of bias was assessed with the JBI checklist for case series. We included 10 full texts describing 192 ML patients. Male gender had the highest detection rate (88%), followed by ulcer of the nasal mucosa (77%), age >15 (69%), and symptom duration >4 months (63%).

**Significance:**

Within this selection of patients, we found that the male gender, ulcer of the nasal mucosa, age >15, and symptom duration >4 months lead to the highest detection rates. However, higher detection comes -naturally- with a higher rate of false positives as well. As we only included ML patients, this could not be verified. Therefore, the criteria that we found to be most promising should be validated in a well-designed prospective study.

## Author summary

Mucosal leishmaniasis, a disease caused by *Leishmania* parasites, is transmitted from animals to humans by sandflies. It is a forgotten disease that affects under-resourced populations in South America. Without treatment, this disease mutilates the face and can even be fatal. Diagnosing mucosal leishmaniasis is challenging. The only available testing in rural areas is the use of either a lesion smear or biopsy for light microscopy, however, this is unreliable. Many patients suffer for years before receiving treatment. Syndromic algorithms use patient characteristics, such as age, gender, and symptoms to identify patients for treatment. This method has been promoted to manage infectious diseases, such as tuberculosis and sexually transmitted diseases, in low resource settings. We explore clinical criteria for a new algorithm to diagnose mucosal leishmaniasis in patients described in the medical literature. We searched the literature for reports written in any language and identified 10 studies describing 192 patients with mucosal leishmaniasis. We found that male gender, ulcer of the nasal mucosa, age >15, and symptom duration >4 months lead to acceptable detection rates. Therefore, diagnostic algorithms might improve the detection of patients with mucosal leishmaniasis but need prospective studies in clinical practice to prove their true potential.

## Introduction

### Background

Mucosal leishmaniasis (ML), a disease caused by the *Leishmania* parasites, is a Neglected Tropical Disease (NTD) that affects under-resourced populations mainly in remote, rural areas of the South American continent [1, 2]. In most cases, ML is caused by *Leishmania braziliensis* and, less commonly by *L*. *guyanensis*, *L*. *panamensis*, or *L*. *amazonensis*. The parasites are transmitted through the bite of an infected female sandfly (genus *Lutzomyia*). Following a bite, most patients develop cutaneous ulcers or nodules, referred to as cutaneous leishmaniasis (CL). ML can develop simultaneously with CL or start months to decades after a healed skin lesion. ML is caused by the dissemination of parasites to the oral, nasal, pharyngeal, and laryngeal mucosa. However, not all new ML patients report a history of CL [3, 4]. Unfortunately, the spontaneous cure of ML is rare. Furthermore, if untreated, ML may progress to nasal septum perforation and destruction, severe facial deformities, airway obstruction, and ultimately, death [5–7]. Antimonials and amphotericin-B are the recommended treatments for ML but both are associated with severe side effects and have to be injected [8–10]. Miltefosine is an expensive systemic agent for oral administration, but has limited efficacy, potentially severe side effects, and is not universally available [9, 11]. Therefore, accurate diagnosis is essential to justify ML treatment. However, diagnosing ML is challenging on clinical grounds alone, as there is a significant number of differential diagnoses such as common rhinitis, chronic sinusitis, banal nasal septum perforation, midline lymphoma, paracoccidioidomycosis, tuberculosis, rhinosporidiosis, nasal scleroma, Wegener´s granulomatosis, histoplasmosis, sporotrichosis, Hansen´s disease, squamous cell carcinoma, and chronic nasal cocaine use, among others [12, 13]. Given the significant harms of not treating ML, a high index of suspicion is warranted in all patients from endemic areas with chronic nasal, oropharyngeal, or laryngeal symptoms. Additionally, serology and the Montenegro skin test can indirectly support the diagnosis of ML, but these are neither sensitive nor specific and often unavailable in rural settings [2, 6, 14, 15]. Therefore, mucosal tissue smear slide or histopathology is recommended for a more precise diagnosis. However, the diagnostic accuracy of both these testing methods is extremely variable but usually low, even when performed in specialized centers, due to the paucity of amastigotes in the mucosal tissue [7, 16, 17]. In addition, even molecular diagnostics, such as polymerase chain reaction techniques, fail to confirm the diagnosis in more than a quarter of patients and are usually unavailable in resource-limited endemic areas [18, 19].

Syndromic algorithms for the diagnosis of infectious diseases have shown effectivity, and are often the only option in resource-limited settings [20–22]. To the best of our knowledge, little evidence exists on the application of syndromic algorithms for ML diagnosis. However, it could increase access to therapy in resource-limited populations. This study explores clinical criteria for syndromic ML diagnosis in low-resource settings in South America. Before designing a prospective diagnostic accuracy study to evaluate a syndromic algorithm, we planned to assess the accuracy of a syndromic algorithm in the existing literature. However, this requires meticulous reporting of patient characteristics and test results, as done in case series and diagnostic accuracy studies (containing patients with and without ML). As no diagnostic accuracy studies of any syndromic algorithm for ML diagnosis were available, we aimed to investigate the ML detection rates of predefined clinical characteristics and test results. Hereto, case series of ML patients were retrieved through a systematic literature review and the presence of predefined characteristics and test results in these case series were recorded.

### Objective

Objective: To explore the ML detection rates of clinical criteria in participants from endemic areas in South America.

## Methods

### Protocol

The protocol for this systematic review was pre-registered on August 10, 2017, in the PROSPERO International prospective register of systematic reviews with registration number: CRD42017074148, 2017 [23] and is available from; https://www.crd.york.ac.uk/prospero/display_record.php?ID=CRD42017074148. This systematic review followed the Preferred Reporting Items for a Systematic Review and Meta-analysis (PRISMA) guideline [24].

### General study eligibility criteria

Study eligibility criteria for inclusion in this systematic review are described in Table 1. Case series were included without date or language limitations. Treatment studies and studies reporting on less than five ML patients were excluded to avoid selection bias.

**Table 1:** General study eligibility criteria for inclusion in this systematic review.

**Inclusion criteria** **1.** Case series with any publication date or language **2.** Report on patients with a history of a stay in an ML epidemic area of South America **3.** Report on patients with active nose, throat, or oral disease (e.g. obstruction, hyperemia, erosion, ulceration, or granulomatous lesions) **4.** Present clinical information at individual patient level. **Exclusion criteria:** **1.** Narrative reviews **2.** Studies reporting on <5 ML patients **3.** Treatment studies **4.** Duplicate publication on the same individual patient or patient group **5.** Patients without a history of a visit to South America

ML: Mucosal Leishmaniasis

### General study identification

PUBMED, EMBASE, Web of Science, SCIELO, and LILACS databases were searched without restrictions, with the last search on the 14^th^ of April 2022. The following search string was applied In PUBMED: ¨human AND (mucocutan* OR mucos* OR mucous OR tegument* OR nasal) AND (leishmanias* OR leishmanios*)¨. The annotation of the search string was adjusted for each literature database. In addition, reference lists of studies included for full-text analysis were searched for additional papers.

### Study selection

Title and abstract screening were performed using the Rayyan QCR software [25]. Full texts of included studies were either retrieved electronically or requested manually by the medical library of the University of Amsterdam. Full texts were assessed using a predefined checklist (S1 Table) and included if they matched the eligibility criteria (Table 1). Each step of the study selection was done independently by JB and either KM or CN. Differences were resolved through consensus, or with help of HdV or HS.

### General data collection

All steps in data collection were individually performed by JB and either KM or CN. Disputes were resolved through consensus. Using a pre-defined form, the following information was extracted from individual patients in the included papers: identifiers of the patient, presence of concomitant CL, diagnostic method(s) used, HIV status, other concomitant illnesses, results of histopathology, and smear slide microscopy (both defined as positive exclusively in case of amastigote visualization), causing *Leishmania* species, medications used before diagnosis, stage of disease according to Lessa *et al*. [6], presenting symptoms; epistaxis, dysphagia or odynophagia, voice changes, CL-scar, ulceration of the nasal mucosa, nasal deformation, oropharyngeal lesions, and symptom duration.

### Risk of bias assessment

Risk of bias assessment was done with the JBI checklist for case series [26]. Because this study retrieved data at the individual patient level, the question on the appropriateness of the statistical analysis was obviated from the JBI checklist. The following question was added to assess the possible risk of bias through the inclusion of a specific patient population: `Did the case series avoid exclusion based on clinical characteristics?’. Risk of bias assessment was individually performed by JB and KM. Disputes were resolved through discussion.

### Data analysis

All included studies were investigated for 12 predefined binary clinical criteria that are frequently mentioned in the literature and are easily available in clinical practice in rural settings, as our ultimate goal is to develop an algorithm for syndromic management (See Table 2). Cumulative detection rates were calculated per patient with Microsoft Excel 2018 software [27]. For cumulative detection rate calculation, the criteria were arranged from the highest absolute number of patients positive to the lowest. Non-reported criteria were interpreted as negative. Cumulative detection rates were calculated separately for males and females to avoid gender-based selection of patients by a diagnostic algorithm. Because of the low quality of the included studies and the exploratory nature of this paper, a meta-analysis was not done.

**Table 2:** Twelve rurally available clinical criteria assessed for diagnostic accuracy in this study.

Criterion	Reason
Male	Risk factor for ML [28]
Age >15 years	Risk factor for ML [28, 29]
Symptom duration >4 months	Differentiation from acute viral syndromes [30]
Ulcer of the nasal mucosa	Present from stage 2 of the disease [6]
Epistaxis	Present from stage 2 of the disease [6]
Oropharyngeal lesion	Worse prognosis of the disease[31]
Dysphagia or odynophagia	Sign of severe disease [6]
Nasal deformation	Sign of severe disease [6]
CL scar	Risk factor for ML [4, 7]
Concomitant CL	Risk factor for ML [4, 32]
Histopathology	Current confirmative test [33]
Smear slide microscopy	Current confirmative test [14]

## Results

### Study selection and data obtained

**Fig 1:**
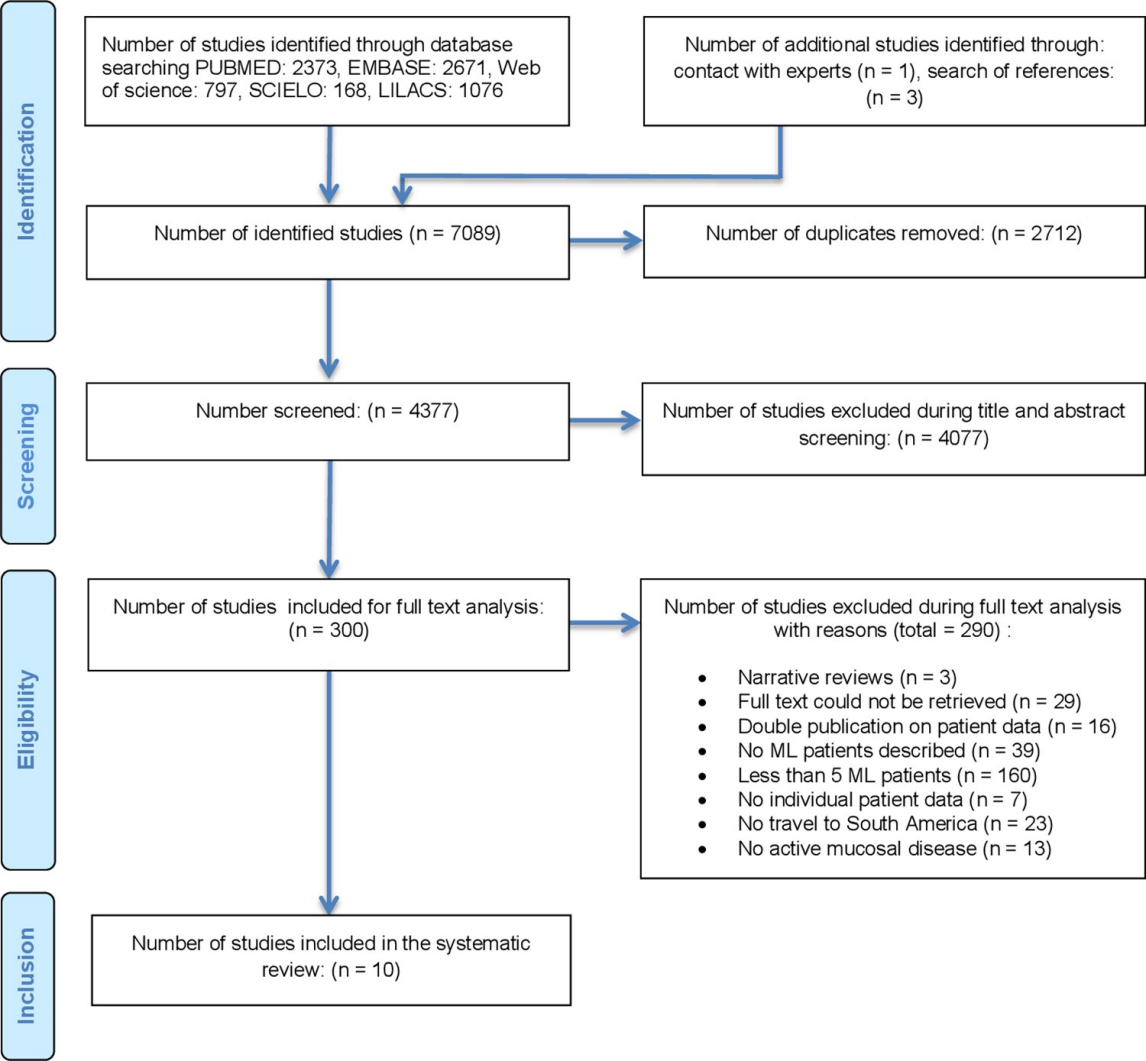
PRISMA literature assessment flow diagram.

After the removal of duplicates, 4377 reports were retrieved through the searches in different databases. Of these, 10 were included that reported on a total of 192 ML patients [5, 6, 16, 34–40]. 160 full texts were excluded because they reported on less than five ML patients and seven because they presented no data at the individual patient level. The reasons for full text exclusions are summarized in Fig 1.

### General study characteristics

This review included 10 case series that were all published between 1968 and 2019. Most studies reported on patients diagnosed in Brazil (n = 6), followed by Peru (n = 2). The median number of reported ML cases per publication was 13, ranging from 5 to 50. Most patients were diagnosed with several methods, including PCR, Montenegro skin test, culture, and histopathology. *L*. *braziliensis* was reported as the most common species in ML lesions. However, the species was unknown in two (20%) of the included studies (Tables 3 and S1).

**Table 3:** Characteristics of the 10 case series reporting on 192 ML patients included in the systematic review.

**Study characteristic**		
Year published (median, range)	2007	1968–2019
Number of ML patients (median, range)	13	5–50
**Country of diagnosis**	**Number of studies**	**Number of patients (%)**
Brazil	6	101 (53)
Peru	2	73 (38)
Ecuador	1	13 (7)
United Kingdom	1	5 (3)
**Diagnostic method for inclusion in study** ^**a**^		
PCR	7	64 (33)
Montenegro skin test	8	115 (60)
Culture	4	22 (11)
Histopathology	9	51 (27)
Serology	3	27 (14)
Smear slide microscopy	1	13 (7)
Cure with antimonial treatment	2	6 (3)
**Reported causative *Leishmania* species** ^**b**^		
*L*. *braziliensis*	8	70 (36)
*L*. *amazonensis*	2	6 (3)
*L*. *viannia* complex	2	12 (6)
Unknown	2	104 (54)

^a^The majority of studies applied several diagnostic methods to every patient

^b^Several studies reported mixed causative species and the species was unknown in a part of the patients.

### Risk of bias assessment

**Fig 2:**
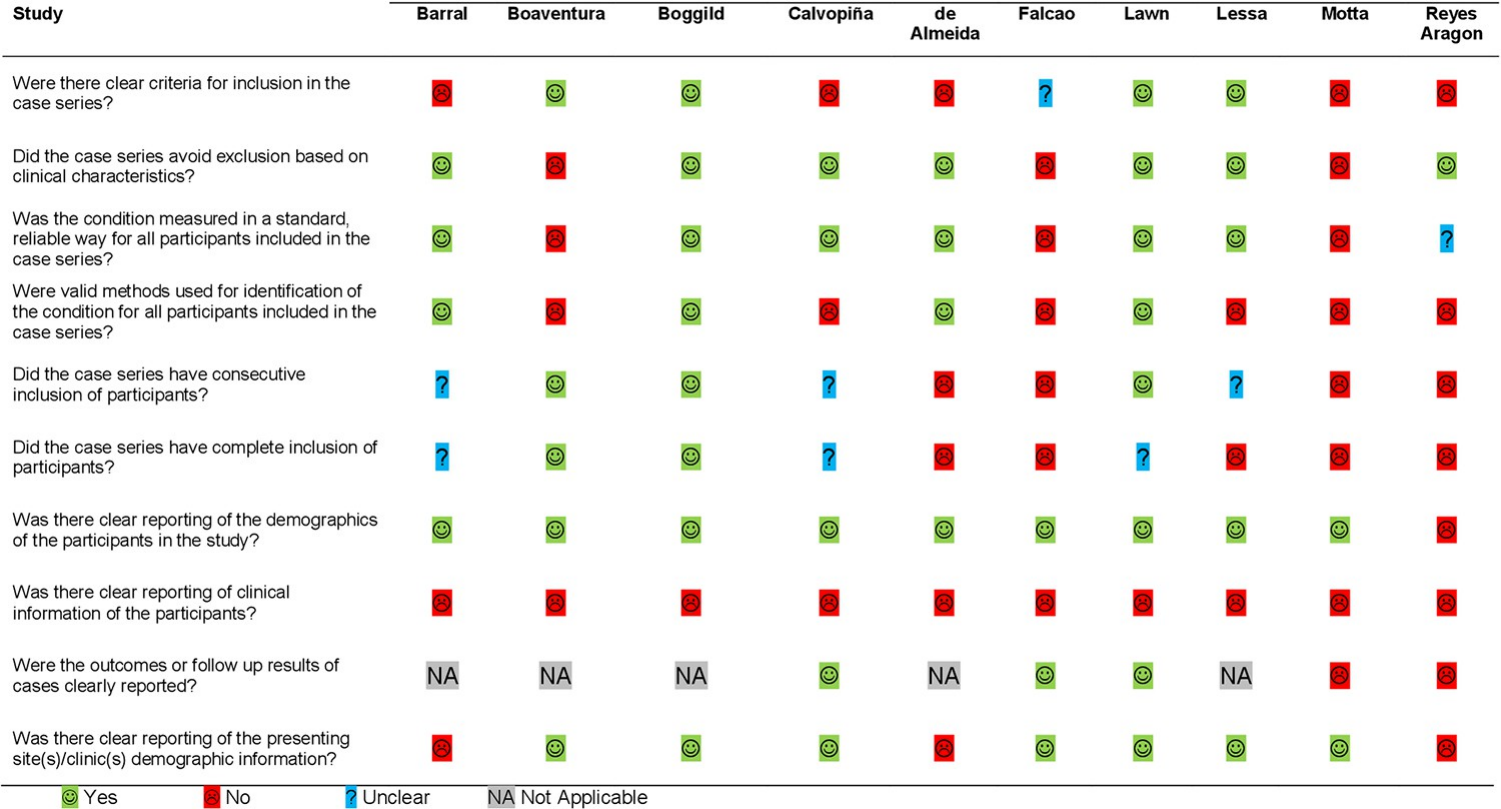
Risk of bias assessment of the included studies with the modified JBI checklist for case series.

Assessment of the risk of bias in the 10 case series included in this systematic review, using the modified JBI checklist for case series, revealed a high risk of bias in all the included studies. Six studies lacked clear inclusion criteria and three studies included patients on the basis of clinical characteristics: Boaventura *et al*. included patients if they had concomitant CL and Falcao *et al*. and Motta *et al*. included patients if they had oral lesions. Four studies did not describe ML diagnostic methods clearly. Six studies applied indirect methods (skin test, serology, or cure on antimonial treatment) for ML diagnosis. Only two studies included patients consecutively and completely. Patient demographics (age, gender, and duration of symptoms) were unclearly described by one study. No study reported completely on the clinical criteria explored for ML detection rates. Follow-up was unclear in two studies and demographic data of the study site was unclear in three. Results of the risk of bias assessment are summarized in Fig 2.

### Findings

All of the study participants’ genders were known, with 88% of them being male. Information was incomplete for the other criteria. The male gender resulted in the highest number of patients positive, followed by ulcer of the nasal mucosa, age >15, and symptom duration >4 months. Results of histopathology and smear slide microscopy were unknown for the majority of patients and were positive in 55 and 41% of reported patients respectively (Table 4).

**Table 4:** Arrangement of the clinical criteria from the highest absolute number of patients positive to the lowest.

Nr.	Criterion	Reported in N patients (%)	N positive (% of reported)	Detection rate
1	Male	192 (100)	168 (88)	0,88
2	Ulcer of the nasal mucosa	159 (83)	147 (92)	0,77
3	Age>15	141 (73)	133 (94)	0,69
4	Symptom duration >4 months	148 (77)	121 (82)	0,63
5	Oropharyngeal lesion	155 (81)	88 (57)	0,46
6	Epistaxis	63 (33)	58 (92)	0,30
7	Histopathology	93 (48)	51 (55)	0,27
8	Dysphagia or odynophagia	42 (22)	40 (95)	0,21
9	CL scar	57 (30)	36 (63)	0,19
10	Concomitant CL	68 (35)	31 (46)	0,16
11	Nasal deformation	86 (45)	30 (34)	0,16
12	Smear slide microscopy	32 (17)	13 (41)	0,07

N = Number

The cumulative detection rates of clinical criteria for males and females are shown in Tables 5 and 6. Two or more positives out of the three criteria `ulcer of the nasal mucosa’, `age >15’, and `symptom duration >4 months’ had a cumulative detection rate of 84% in males and 79% in females. Three or more positives out of the six criteria `ulcer of the nasal mucosa’, `age >15’, `symptom duration >4 months’, `oropharyngeal lesion’, `epistaxis’, and `histopathology positive’ had a cumulative detection rate of 75% in males and 54% in females.

**Table 5:** Cumulative detection rates of clinical criteria in 168 male Mucosal Leishmaniasis (ML) patients.

Nr.	Criterion	Cumulative detection rates at individual patient level			
	Cut-off score	≥1	≥2	≥3	≥4
2	Ulcer of the nasal mucosa (%)	139 (83)	0 (0)	0 (0)	0 (0)
3	Age>15 (%)	160 (95)	80 (48)	0 (0)	0 (0)
4	Symptom duration >4 months (%)	168 (100)	141 (84)	42 (25)	0 (0)
5	Oropharyngeal lesion (%)	168 (100)	162 (96)	88 (52)	14 (8)
6	Epistaxis (%)	168 (100)	163 (97)	104 (62)	40 (24)
7	Histopathology positive (%)	168 (100)	163 (97)	126 (75)	60 (36)
8	Dysphagia or odynophagia (%)	168 (100)	163 (97)	130 (77)	75 (45)
9	CL-scar (%)	168 (100)	164 (98)	144 (86)	82 (49)
10	Concomitant CL (%)	168 (100)	165 (98)	148 (88)	88 (52)
11	Nasal deformation (%)	168 (100)	165 (98)	153 (91)	99 (59)
12	Smear slide microscopy positive (%)	168 (100)	165 (98)	153 (91)	102 (61)

**Table 6:** Cumulative detection rates of clinical criteria in 24 female Mucosal Leishmaniasis (ML) patients.

Nr.	Criterion	Cumulative detection rates at individual patient level			
	Cut-off score	≥1	≥2	≥3	≥4
2	Ulcer of the nasal mucosa (%)	18 (75)	0 (0)	0 (0)	0 (0)
3	Age>15 (%)	24 (100)	16 (67)	0 (0)	0 (0)
4	Symptom duration >4 months (%)	24 (100)	19 (79)	7 (29)	0 (0)
5	Oropharyngeal lesion (%)	24 (100)	22 (92)	8 (33)	3 (13)
6	Epistaxis (%)	24 (100)	23 (96)	9 (38)	6 (25)
7	Histopathology positive (%)	24 (100)	23 (96)	13 (54)	6 (25)
8	Dysphagia or odynophagia (%)	24 (100)	23 (96)	14 (58)	7 (29)
9	CL-scar (%)	24 (100)	23 (96)	18 (75)	7 (29)
10	Concomitant CL (%)	24 (100)	23 (96)	20 (83)	11 (46)
11	Nasal deformation (%)	24 (100)	23 (96)	20 (83)	11 (46)
12	Smear slide microscopy positive (%)	24 (100)	23 (96)	20 (83)	11 (46)

## Discussion

The objective of this study was to explore the ML detection rates of clinical criteria combinations. In our systematic review, we included 10 case series reporting on more than 190 ML patients in South America.

Our main finding is the acceptable ML detection rate of clinical criteria and promising combinations for ML diagnostic algorithms. As accurate reference tests are often unavailable in lower resource settings, such as many centers in South America [14, 15], algorithms for syndromic diagnosis for ML would be highly desirable to select patients for the, often toxic, treatment.

Adverse effects, such as musculoskeletal pain and gastrointestinal disturbances, are very common after administration of antimonials and severe complications such as arrhythmias, leucopenia, hepatitis, and pancreatitis occur in up to 14% of treated patients. Occasionally, even deaths are reported from patients under antimonial treatment [8]. To avoid drug toxicities in patients who do not have ML, it is at least as important to know the proportion of false positive results as the proportion of true positives. However, the proportion of false positives (one minus the specificity) requires a study sample including participants without ML and these were not included in this study. Therefore, we have no estimates of the specificity of clinical criteria combinations and we cannot rule out that the proportion of false positive results of syndromic algorithms may be too high.

The absence of an established universal reference test for ML diagnosis limits the current study. We included ML patients diagnosed with any of the currently applied tests in South America, including the Montenegro skin test, serology, and cure with antimonial treatment. This results in the possible inclusion of non-ML patients in this study and the overestimation of detection rates.

Risk of bias evaluation revealed significant flaws in all the studies leading to a high risk of bias. The incomplete reporting of clinical criteria limits this study and leads to a possible underestimation of the detection rates as non-reported criteria were interpreted as negative. The selection of concomitant CL or oral ML patients by three studies possibly leads to an overestimation of the detection rates of concomitant CL and oropharyngeal lesions.

Former studies have reported that the male gender is a risk factor for leishmaniasis in its cutaneous (CL), mucosal (ML), and visceral expressions [28, 41]. The underlying processes could be sex-specific biological, differences in vector exposure, variances in health-seeking behavior, and marginalization of female patients in health care and publications [42–44]. Therefore, it is not unexpected that the majority of the patients in this study are men. As the methodological quality of the included studies is low, we cannot exclude that the gender difference was at least partially caused by biased patient inclusion or publication. That also applies to the criterion `age >15’. We emphasize that the clinical criteria combinations shown in this paper have to be adapted according to the results of a well-designed prospective study (including non-ML patients) that should avoid the exclusion of patients based on gender, age, ethnicity, and other personal identifiers and include an evaluation of health-seeking behavior in its protocol.

The ML detection rates of clinical criteria combinations explored in this review reached levels comparable to the performance of the Montenegro skin test [7, 45]. Their application would be rapid, cheap, and feasible in any rural clinical setting located in endemic regions and thus of potential clinical value.

## Conclusion

We present an exploration of the detection rates of clinical criteria in 192 ML patients reported in case series. Within this selection of patients, we found that male gender, ulcer of the nasal mucosa, age >15, and symptom duration >4 months lead to the highest detection rates. They could improve diagnosis and hence prompt treatment of ML in vulnerable groups in resource-limited settings where diagnostic confirmation cannot be obtained. Therefore, the criteria that we found to be most promising, should be validated in a well-designed prospective study.

## Supporting information

S1 Appendix PRISMA 2020 abstract checklist for systematic reviews. (DOCX)

S2 Appendix PRISMA 2020 checklist for systematic reviews. (DOCX)

S1 Table Characteristics of included studies. (XLSX)

S2 Table Characteristics of 192 individually assessed patients. (XLSX)
