# Supplementary material for: Clinical criteria for Mucosal Leishmaniasis diagnosis in rural South America: A systematic literature review
Source: PLoS Negl Trop Dis. 2022 Aug 10;16(8):e0010621. doi: 10.1371/journal.pntd.0010621 (PMC9365133; doi:10.1371/journal.pntd.0010621)
Supplement: S1 Appendix — (DOCX) [file pntd.0010621.s001.docx]

| **Section and Topic** | **Item #** | **Checklist item** | **Reported (Yes/No)** |
| --- | --- | --- | --- |
| **TITLE** | | |  |
| Title | 1 | Identify the report as a systematic review.  *Clinical criteria for Mucosal Leishmaniasis diagnosis in rural South America: a systematic literature review.* | Line 4 |
| **BACKGROUND** | | |  |
| Objectives | 2 | Provide an explicit statement of the main objective(s) or question(s) the review addresses.  *We explore potential clinical criteria for a syndromic diagnostic algorithm for ML in rural healthcare settings in South America.* | Line 48 |
| **METHODS** | | |  |
| Eligibility criteria | 3 | Specify the inclusion and exclusion criteria for the review.  *Any original, non-treatment study was eligible, and case reports were excluded.* | Line 54 |
| Information sources | 4 | Specify the information sources (e.g. databases, registers) used to identify studies and the date when each was last searched.  *PUBMED, EMBASE, Web of Science, SCIELO, and LILACS databases were searched without restrictions.* | Line 55 |
| Risk of bias | 5 | Specify the methods used to assess risk of bias in the included studies.  *The risk of bias was assessed with the JBI checklist for case series.* | Line 56 |
| Synthesis of results | 6 | Specify the methods used to present and synthesise results.  *we explored the cumulative ML detection rates of clinical criteria* | Line 53 |
| **RESULTS** | | |  |
| Included studies | 7 | Give the total number of included studies and participants and summarise relevant characteristics of studies.  *We included 10 full texts describing 192 ML patients.* | Line 57 |
| Synthesis of results | 8 | Present results for main outcomes, preferably indicating the number of included studies and participants for each. If meta-analysis was done, report the summary estimate and confidence/credible interval. If comparing groups, indicate the direction of the effect (i.e. which group is favoured).  *Male gender had the highest detection rate (88%), followed by ulcer of the nasal mucosa (77%), age >15 (69%), and symptom duration >4 months (63%).* | Line 57 |
| **DISCUSSION** | | |  |
| Limitations of evidence | 9 | Provide a brief summary of the limitations of the evidence included in the review (e.g. study risk of bias, inconsistency and imprecision).  *However, higher detection comes -naturally- with a higher rate of false positives as well. As we only included ML patients, this could not be verified.* | Line 62 |
| Interpretation | 10 | Provide a general interpretation of the results and important implications.  *Therefore, the criteria that we found to be most promising should be validated in a well-designed prospective study.* | Line 64 |
| **OTHER** | | |  |
| Funding | 11 | Specify the primary source of funding for the review.  *This is reported separately for PLOS NTD:*  *JB received a monthly volunteer allowance from Latin Link Nederland http://www. latinlink-nederland.nl/, which helped fund the study. Latin Link had no role in study design, data collection and analysis, decision to publish, preparation of the manuscript. No additional external funding was received for this study.* |  |
| Registration | 12 | Provide the register name and registration number.  *The protocol for this systematic review was pre-registered in PROSPERO with the number: CRD42017074148.* | Line 51 |

*From:*  Page MJ, McKenzie JE, Bossuyt PM, Boutron I, Hoffmann TC, Mulrow CD, et al. The PRISMA 2020 statement: an updated guideline for reporting systematic reviews. BMJ 2021;372:n71. doi: 10.1136/bmj.n71

For more information, visit: <http://www.prisma-statement.org/>
