# Supplementary material for: Clinical criteria for Mucosal Leishmaniasis diagnosis in rural South America: A systematic literature review
Source: PLoS Negl Trop Dis. 2022 Aug 10;16(8):e0010621. doi: 10.1371/journal.pntd.0010621 (PMC9365133; doi:10.1371/journal.pntd.0010621)
Supplement: S2 Appendix — (DOCX) [file pntd.0010621.s002.docx]

|  | **Item #** | **Checklist item** | **Location where item is reported** |
| --- | --- | --- | --- |
| **TITLE** | | |  |
| Title | 1 | Identify the report as a systematic review.  *Clinical criteria for Mucosal Leishmaniasis diagnosis in rural South America: a systematic literature review.* | Line 4 |
| **ABSTRACT** | | |  |
| Abstract | 2 | *See S1_Appendix* | Line 442 |
| **INTRODUCTION** | | |  |
| Rationale | 3 | Describe the rationale for the review in the context of existing knowledge.  *Therefore, accurate diagnosis is essential to justify ML treatment. However, diagnosing ML is challenging on clinical grounds alone, as there is a significant number of differential diagnoses* | Line 102 |
| Objectives | 4 | Provide an explicit statement of the objective(s) or question(s) the review addresses.  *Objective: To explore the ML detection rates of clinical criteria in participants from endemic areas in South America.* | Line 136 |
| **METHODS** | | |  |
| Eligibility criteria | 5 | Specify the inclusion and exclusion criteria for the review and how studies were grouped for the syntheses.  ***Table 1. General study eligibility criteria for inclusion in this systematic review.*** | Line 152 |
| Information sources | 6 | Specify all databases, registers, websites, organisations, reference lists and other sources searched or consulted to identify studies. Specify the date when each source was last searched or consulted.  *PUBMED, EMBASE, Web of Science, SCIELO, and LILACS databases were searched without restrictions, with the last search on the 14^th^ of April 2022.* | Line 156 |
| Search strategy | 7 | Present the full search strategies for all databases, registers and websites, including any filters and limits used.  *The following search string was applied In PUBMED: ¨human AND (mucocutan* OR mucos* OR mucous OR tegument* OR nasal) AND (leishmanias* OR leishmanios*)¨. The annotation of the search string was adjusted for each literature database.* | Line 157 |
| Selection process | 8 | Specify the methods used to decide whether a study met the inclusion criteria of the review, including how many reviewers screened each record and each report retrieved, whether they worked independently, and if applicable, details of automation tools used in the process.  *Title and abstract screening were performed using the Rayyan QCR software [*[*25*](#_ENREF_25)*]. Full texts of included studies were either retrieved electronically or requested manually by the medical library of the University of Amsterdam. Full texts were assessed using a predefined checklist (S1 Table) and included if they matched the eligibility criteria (Table 1). Each step of the study selection was done independently by JB and either KM or CN.* | Line 163 |
| Data collection process | 9 | Specify the methods used to collect data from reports, including how many reviewers collected data from each report, whether they worked independently, any processes for obtaining or confirming data from study investigators, and if applicable, details of automation tools used in the process.  *All steps in data collection were individually performed by JB and either KM or CN. Disputes were resolved through consensus. Using a pre-defined form, the following information was extracted from individual patients in the included papers:* | Line 170 |
| Data items | 10a | List and define all outcomes for which data were sought. Specify whether all results that were compatible with each outcome domain in each study were sought (e.g. for all measures, time points, analyses), and if not, the methods used to decide which results to collect.  *identifiers of the patient, presence of concomitant CL, diagnostic method(s) used, HIV status, other concomitant illnesses, results of histopathology, and smear slide microscopy (both defined as positive exclusively in case of amastigote visualization),* | Line 172 |
|  | 10b | List and define all other variables for which data were sought (e.g. participant and intervention characteristics, funding sources). Describe any assumptions made about any missing or unclear information.  *Full texts were assessed using a predefined checklist (S1 Table)* | Line 165 |
| Study risk of bias assessment | 11 | Specify the methods used to assess risk of bias in the included studies, including details of the tool(s) used, how many reviewers assessed each study and whether they worked independently, and if applicable, details of automation tools used in the process.  *Risk of bias assessment was done with the JBI checklist for case series [*[*26*](#_ENREF_26)*].* | Line 180 |
| Effect measures | 12 | Specify for each outcome the effect measure(s) (e.g. risk ratio, mean difference) used in the synthesis or presentation of results.  *Cumulative detection rates were calculated per patient with Microsoft Excel 2018 software [*[*27*](#_ENREF_27)*].* | Line 189 |
| Synthesis methods | 13a | Describe the processes used to decide which studies were eligible for each synthesis (e.g. tabulating the study intervention characteristics and comparing against the planned groups for each synthesis (item #5)).  *This does not apply to our manuscript as all patients were included in the synthesis.* |  |
|  | 13b | Describe any methods required to prepare the data for presentation or synthesis, such as handling of missing summary statistics, or data conversions.  *Non-reported criteria were interpreted as negative.* | Line 192 |
|  | 13c | Describe any methods used to tabulate or visually display results of individual studies and syntheses.  *Cumulative detection rates were calculated separately for males and females to avoid gender-based selection of patients by a diagnostic algorithm.* | Line 193 |
|  | 13d | Describe any methods used to synthesize results and provide a rationale for the choice(s). If meta-analysis was performed, describe the model(s), method(s) to identify the presence and extent of statistical heterogeneity, and software package(s) used.  *All included studies were investigated for 12 predefined binary clinical criteria that are frequently mentioned in the literature and are easily available in clinical practice in rural settings, as our ultimate goal is to develop an algorithm for syndromic management (See table 2).* | Line 187 |
|  | 13e | Describe any methods used to explore possible causes of heterogeneity among study results (e.g. subgroup analysis, meta-regression).  *Because of the low quality of the included studies and the exploratory nature of this paper, a meta-analysis was not done.* | Line 194 |
|  | 13f | Describe any sensitivity analyses conducted to assess robustness of the synthesized results.  *Because of the low quality of the included studies and the exploratory nature of this paper, a meta-analysis was not done.* | Line 194 |
| Reporting bias assessment | 14 | Describe any methods used to assess risk of bias due to missing results in a synthesis (arising from reporting biases).  *Because of the low quality of the included studies and the exploratory nature of this paper, a meta-analysis was not done.* | Line 194 |
| Certainty assessment | 15 | Describe any methods used to assess certainty (or confidence) in the body of evidence for an outcome.  *Because of the low quality of the included studies and the exploratory nature of this paper, a meta-analysis was not done.* | Line 194 |
| **RESULTS** | | |  |
| Study selection | 16a | Describe the results of the search and selection process, from the number of records identified in the search to the number of studies included in the review, ideally using a flow diagram.  *After the removal of duplicates, 4377 reports were retrieved through the searches in different databases.* | Line 201 |
|  | 16b | Cite studies that might appear to meet the inclusion criteria, but which were excluded, and explain why they were excluded.  *The reasons for full text exclusions are summarized in Fig 1.* | Line 204 |
| Study characteristics | 17 | Cite each included study and present its characteristics.  *Of these, 10 were included that reported on a total of 192 ML patients [*[*5*](#_ENREF_5)*,* [*6*](#_ENREF_6)*,* [*16*](#_ENREF_16)*,* [*34-40*](#_ENREF_34)*].* | Line 202 |
| Risk of bias in studies | 18 | Present assessments of risk of bias for each included study.  ***Fig 2. Risk of bias assessment of the included studies with the modified JBI checklist for case series.*** | Line 237 |
| Results of individual studies | 19 | For all outcomes, present, for each study: (a) summary statistics for each group (where appropriate) and (b) an effect estimate and its precision (e.g. confidence/credible interval), ideally using structured tables or plots.  ***Table 4. Arrangement of the clinical criteria from the highest absolute number of patients positive to the lowest.***  ***S1 Table. Characteristics of included studies*** | Line 247 and 445 |
| Results of syntheses | 20a | For each synthesis, briefly summarise the characteristics and risk of bias among contributing studies.  *Assessment of the risk of bias in the 10 case series included in this systematic review, using the modified JBI checklist for case series, revealed a high risk of bias in all the included studies.* | Line 225 |
|  | 20b | Present results of all statistical syntheses conducted. If meta-analysis was done, present for each the summary estimate and its precision (e.g. confidence/credible interval) and measures of statistical heterogeneity. If comparing groups, describe the direction of the effect.  *Because of the low quality of the included studies and the exploratory nature of this paper, a meta-analysis was not done.* | Line 194 |
|  | 20c | Present results of all investigations of possible causes of heterogeneity among study results.  *Because of the low quality of the included studies and the exploratory nature of this paper, a meta-analysis was not done.* | Line 194 |
|  | 20d | Present results of all sensitivity analyses conducted to assess the robustness of the synthesized results.  *Because of the low quality of the included studies and the exploratory nature of this paper, a meta-analysis was not done.* | Line 194 |
| Reporting biases | 21 | Present assessments of risk of bias due to missing results (arising from reporting biases) for each synthesis assessed.  ***Reported in N patients (%)*** | Line 248 |
| Certainty of evidence | 22 | Present assessments of certainty (or confidence) in the body of evidence for each outcome assessed.  *Because of the low quality of the included studies and the exploratory nature of this paper, a meta-analysis was not done.* | Line 194 |
| **DISCUSSION** | | |  |
| Discussion | *23a* | *Provide a general interpretation of the results in the context of other evidence.*  *Our main finding is the acceptable ML detection rate of clinical criteria and promising combinations for ML diagnostic algorithms.* | Line 268 |
|  | 23b | Discuss any limitations of the evidence included in the review.  *Therefore, we have no estimates of the specificity of clinical criteria combinations* | Line 279 |
|  | 23c | Discuss any limitations of the review processes used.  *The absence of an established universal reference test for ML diagnosis limits the current study.* | Line 282 |
|  | 23d | Discuss implications of the results for practice, policy, and future research.  *Their application would be rapid, cheap, and feasible in any rural clinical setting located in endemic regions and thus of potential clinical value.* | Line 303 |
| **OTHER INFORMATION** | | |  |
| Registration and protocol | 24a | Provide registration information for the review, including register name and registration number, or state that the review was not registered.  *in the PROSPERO International prospective register of systematic reviews with registration number: CRD42017074148, 2017 [*[*23*](#_ENREF_23)*] and is available from;* | Line 141 |
|  | 24b | Indicate where the review protocol can be accessed, or state that a protocol was not prepared.  *available from;* [*https://www.crd.york.ac.uk/prospero/display_record.php?ID=CRD42017074148*](https://www.crd.york.ac.uk/prospero/display_record.php?ID=CRD42017074148)*.* | Line 144 |
|  | 24c | Describe and explain any amendments to information provided at registration or in the protocol.  [*https://www.crd.york.ac.uk/prospero/display_record.php?ID=CRD42017074148*](https://www.crd.york.ac.uk/prospero/display_record.php?ID=CRD42017074148) | Line 144 |
| Support | 25 | Describe sources of financial or non-financial support for the review, and the role of the funders or sponsors in the review.  *This is reported separately for PLOS NTD:*  *JB received a monthly volunteer allowance from Latin Link Nederland http://www. latinlink-nederland.nl/, which helped fund the study. Latin Link had no role in study design, data collection and analysis, decision to publish, preparation of the manuscript. No additional external funding was received for this study.* |  |
| Competing interests | 26 | Declare any competing interests of review authors.  *Non declared* |  |
| Availability of data, code and other materials | 27 | Report which of the following are publicly available and where they can be found: template data collection forms; data extracted from included studies; data used for all analyses; analytic code; any other materials used in the review.  ***S1 Table. Characteristics of included studies***  ***S2 Table. Characteristics of 192 individually assessed patients*** | Line 444 |

*From:*  Page MJ, McKenzie JE, Bossuyt PM, Boutron I, Hoffmann TC, Mulrow CD, et al. The PRISMA 2020 statement: an updated guideline for reporting systematic reviews. BMJ 2021;372:n71. doi: 10.1136/bmj.n71

For more information, visit: <http://www.prisma-statement.org/>
